# Work related injuries: estimating the incidence among illegally employed immigrants

**DOI:** 10.1186/1756-0500-3-331

**Published:** 2010-12-08

**Authors:** Giuseppe Mastrangelo, Ragnar Rylander, Alessandra Buja, Gianluca Marangi, Emanuela Fadda, Ugo Fedeli, Luca Cegolon

**Affiliations:** 1 Padua University, Department of Environmental Medicine and Public Health, Padua, Italy; 2 BioFact Environmental Health Research Center, Lerum, Sweden; 3 PCT 20, Health and Safety at Work Department, Verona, Veneto Region, Italy; 4 Regional Epidemiology Service (SER), Castelfranco Veneto, Italy; 5 Imperial College London, School of Public Health, St. Mary's Campus, Norfolk Place, London, UK

## Abstract

**Background:**

Statistics on occupational accidents are based on data from registered employees. With the increasing number of immigrants employed illegally and/or without regular working visas in many developed countries, it is of interest to estimate the injury rate among such unregistered workers.

**Findings:**

The current study was conducted in an area of North-Eastern Italy. The sources of information employed in the present study were the Accidents and Emergencies records of a hospital; the population data on foreign-born residents in the hospital catchment area (Health Care District 4, Primary Care Trust 20, Province of Verona, Veneto Region, North-Eastern Italy); and the estimated proportion of illegally employed workers in representative samples from the Province of Verona and the Veneto Region. Of the 419 A&E records collected between January and December 2004 among non European Union (non-EU) immigrants, 146 aroused suspicion by reporting the home, rather than the workplace, as the site of the accident. These cases were the numerator of the rate. The number of illegally employed non-EU workers, denominator of the rate, was estimated according to different assumptions and ranged from between 537 to 1,338 individuals. The corresponding rates varied from 109.1 to 271.8 per 1,000 non-EU illegal employees, against 65 per 1,000 reported in Italy in 2004.

**Conclusions:**

The results of this study suggest that there is an unrecorded burden of illegally employed immigrants suffering from work related injuries. Additional efforts for prevention of injuries in the workplace are required to decrease this number. It can be concluded that the Italian National Institute for the Insurance of Work Related Injuries (INAIL) probably underestimates the incidence of these accidents in Italy.

## Background

The absolute number of adult immigrants with a regular visa in Italy was approximately 2,300,000 in 2004 [1]. In the same year, 1,800,000 immigrants had work-related health insurance [2].

According to the National Institute for the Insurance of Work Related Injuries (INAIL, Italian acronym), the number of work related accidents among foreign-born employees was nearly 116,000 in 2004. The corresponding incidence rate was approximately 65 injuries per 1,000 insured persons as compared with 40 among all workers [2]. These statistics are, however, based on information supplied by employers on subjects with legal employment positions. This study aims to estimate the rate of work-related accidents among employees without a legal employment position and/or regular working visa in an area of North Eastern Italy, as the number of these individuals is reported to be increasing in many countries [3].

The present study adopts a more accurate methodological approach to update the findings of a previously published analysis based on some of the data analysed here. In particular, despite using the same numerator of the injury rate, the present study uses an estimate of the denominator which is more precise than that employed in the previous paper [4].

## Methods

### Numerator of the accident rate

The source of information was the patients' records from the Accident and Emergency (A&E) Department of San Bonifacio Hospital, located in the Health Care District (HCD) 4, Primary Care Trust (PCT) 20, in the Province of Verona, Veneto Region, North-Eastern Italy. This area has a large number of small/medium size industrial plants and agricultural activities. We selected all the A&E discharge forms (n = 419) between January and December 2004 belonging to migrants from non European Union (non-EU) countries, who declared their injury as having occurred in a non-occupational setting. 146 of them raised suspicion because the accident was reported to have happened at home, and the type of injury did not correspond with the actual description.

### Denominator of the accident rate

Several sources were scrutinized:

• tables from the Italian Office for National Statistics (ISTAT, Italian acronym) [5,6] showing: the number of foreigners, aged 15-64 years, residing in the hospital catchment area (presumably the HCD 4) in mid 2004 (***A***); and the percentage of employed workers among immigrants in North-Eastern Italy in 2006 (***B_1_***). This percentage was set at 100% in a separate estimation (***B_2_***);

• the reports of inspections of workplaces in 2004 [7] providing the proportion of irregularity (number of foreign workers with illegal employment status and/or illegal immigration status divided by the number of legally employed immigrants) in representative samples from the Province of Verona (***C***) and the whole of Veneto (***D***).

Four different estimates of illegally employed immigrants in this area were obtained:

• estimate_1 _= ***A × B_1 _× C***

• estimate_2 _= ***A × B_2 _× C***

• estimate_3 _= ***A × B_1 _× D***

• estimate_4 _= ***A × B_2 _× D***

### Ethical Considerations

This study was developed in the context of a bigger project approved and approved by the Veneto Region aiming at the surveillance of occupational diseases in the Region. Moreover all data were collected and stored anonymously. For these reasons ethical approval was not required.

## Results

The 146 suspected cases were divided into three groups for descriptive purposes.

• *Group 1 (88 cases)*. Information on the employer was reported although such information was not necessary. In the majority of cases, the injury was industry specific such as foreign body in the cornea - engineering industry; neck strain - textile industry; and hand wound/injury sustained by a glass-bartender.

• *Group 2 (47 cases)*. Although no information on the employer was provided, the nature of the injury (corneal abrasion, hand/foot contusion) suggested it more likely occurred at work rather than in a domestic environment.

• *Group 3 (11 cases)*. Diagnoses were incomplete because the patient never returned after the first medical examination, probably to avoid further enquiries being made as to the circumstances surrounding the accident.

Therefore, the above 146 cases were considered as the numerator of the injury rate. Table 1 shows the distribution of injuries according to the body site and the type of lesion.

**Table 1:** 146 cases of injuries by type and location.

	Location of injury					
	
Type of injury	Head (excluding eyes*)	Trunk	Upper or lower limb	Hand or wrist	Foot or ankle	Total
Wound (cut, abrasion, other wounds)	5	0	3	28	7	43
Contusion, concussion, sprain	5	11	15	9	11	51
Fracture	0	1	0	3	2	6
	10	12	18	40	20	100

* Eyes = 46 (abrasion = 9; presence of a foreign body = 17; conjunctivitis = 20)

In the hospital catchment area, the foreign residents of working age were 6,409. The employment rate among legal immigrants was 72%. The proportion of illegal employment was 11.6% in the Province of Verona and 20.9% in the whole of Veneto. Estimated numbers of illegally employed immigrants were: 537 (estimate_1_); 744 (estimate_2_); 996 (estimate_3_); and 1,338 (estimate_4_). The corresponding injury rates were: 271.8, 196.2, 151.1 and 109.1 per 1,000 illegal employees.

## Discussion

This study is based on the same source of information presented in a previously published paper (estimating the injury rate among unregistered workers) to determine the estimate of the injury rate of these unregistered workers. We believe the denominators employed in the present study have by far improved the accurateness of the analysis. Particularly, in this study, the number of legally employed immigrants has been provided directly by ISTAT. Whereas, in the previous study, this number was estimated on the basis of the whole population of the HCD 4 and the percentages of legally employed immigrants as well as on residents aged 15-64 years in a Municipality of the same area. Furthermore in our study the number of foreign workers without a regular working visa or with illegal employment status was estimated according to the percentage of illegally employed immigrants specifically in the Veneto Region and the Province of Verona (part of the Veneto Region). By contrast, the study by Marchiori et al. obtained these estimates on the basis of the percentage of illegally employed immigrants of North-Eastern Italy (a vast area including three different Regions: Veneto; Friuli Venezia Giulia; Trentino Alto Adige) [4].

There are, however, some methodological shortcomings to consider.

Although the classification was made by physicians with substantial experience in occupational medicine, the judgment on workplace versus home related injuries was subjective.

Furthermore, a misclassification cannot be excluded. An under-reporting is possible since an illegal worker who claims his work-related injury as being a domestic accident can be treated at a health care centre that is not an "emergency" department associated with a hospital; rather, a non-reporting clinic or office. Moreover underreporting of work-related injury or illness is also an area of concern for immigrants. It is hypothesized that immigrant workers are reluctant to complain to their employers due to fear of losing their jobs, and employers hiring huge number of immigrants, especially if unregistered, might be less likely to report their workplace injuries. Finally it may be more difficult to verify a death as work-related for an immigrant worker, especially for an unregistered immigrant [8,9]. In addition, the governmental insurance system in Italy may also play a role regarding the underreporting of accidents, especially for illegal workers. In the case of non-occupational disease or injury, 100% of the insurance payout is paid immediately, whereas in case of a workplace related injury, 40% is paid immediately and the remaining 60% is paid once the final diagnosis is established. This might constitute an incentive for over-reporting accidents at home rather than at the workplace. Since both over- and under-estimation are possible, the two opposite misclassifications may have cancelled each other out, although the relative extent is unknown. In calculating the denominator of the rate, different assumptions were adopted to provide a wide range that could include the correct estimate. Therefore, although some uncertainties in these calculations cannot be excluded, we believe that the final estimates are sound and more accurate than those reported by a previous study on the same issue [4].

The accident rate among illegal workers was higher than the 65 per 1,000 reported by INAIL among legally employed non-EU immigrants [2]. One reason is probably the types of industries where these illegal employees work - namely in the engineering industry, building industry and agriculture - where work-related injuries are more common due to the higher risk tasks that are generally performed in these occupations. Furthermore, foreign-born workers are more likely to take risks or be forced to carry out particularly risky tasks and accept longer work shifts as compared to legal workers [8,9]. This can be reasonably applied to immigrants employed illegally too. In fact unregistered workers are normally less prepared for work conditions and thus they might have a lower risk awareness than registered workers, given also that occupational safety training may not be conducted in their primary language [8-11].

To the best of our knowledge, no similar studies exist for a comparison.

## Conclusions

The results of the present study suggest that there is currently an unrecorded burden of illegally employed immigrants suffering from work related injuries. Additional preventive and regulatory efforts in the workplace are required in order to reduce this number. INAIL probably underestimate the incidence of work related injuries in Italy.

## Competing interests

The authors declare that they have no competing interests.

## Authors' contributions

GM conceived the study and performed the statistical analysis, LC and RR drafted the paper, GM contributed to the development of the idea, UF and AB provided epidemiological support. All authors read and approved the final manuscript.
